# Ancient Methods of Treatment

**Published:** 1906-08-25

**Authors:** 


### Ancient Methods of Treatment.

### THE MANAGEMENT OF AN ENGLISH LEPER HOUSE.

This disease is still among us. In London, at the Tropical School of Medicine, Woolwich, a case is now under treatment.

There are few diseases which have disappeared so completely with the advance of civilisation as leprosy. It was a real scourge in Europe during the eleventh and twelfth centuries when, making due allowance for errors in diagnosis, it seems to have attained a frequency of 40 in 10,000, and affected the rich as well as the poor—King Baldwin and the meanest peasant. Leprosy throughout the middle ages was looked upon as a divine visitation, and was therefore a supernatural disease. It appealed strongly to the popular imagination, and the establishment of a leper house was a favourite charitable bequest. Leper houses, therefore, became very numerous in England, and reached their highest development in the eleventh century. The disease then became less common, until at the Reformation it was found that many of the leper hospitals remained as asylums for the blind, whilst others subsequently became "locks."

The Sherburn Hospital, near Durham, was one of the most richly endowed charities in the North of England, and was certainly the largest leper hospital in this country. It was founded by Hugh Pudsey, Bishop of Durham, about 1184, and Dr. George Newman gives the following interesting account of its domestic economy in his excellent essay "On the History of the Decline and Final Extinction of Leprosy as an Endemic Disease in the British Islands." He says: The daily allowance of the lepers was a loaf weighing five marks and a gallon of ale apiece; and between every two lepers one mess or commons of flesh three days in the week; and if fish, then cheese or butter on the remaining four days. There was a double mess on high festivals, and in particular on the feast of St. Cuthbert in Lent, fresh salmon if it could be had, if not then other fresh fish; and on Michaelmas Day four lepers messed on one goose. With fresh fish, flesh, or eggs a measure of salt was delivered. When fresh fish could not be had, red herrings were served three to a single mess, or cheese and butter by weight or three eggs. During Lent each had a razer of wheat to make frumenty and two razers of beans to boil; sometimes greens or onions; and every day, except Sunday, the seventh part of a razer of bean meal, but on Sunday a measure and a half of pulse to make gruel. Red herrings were prohibited from Pentecost to Michaelmas; at the latter each received two razers of apples.

The lepers shared a common kitchen, a common cook and utensils, and firing for cooking, etc.—namely, a lead, two brazen pots, a table, a large wooden vessel for washing or making wine, a laver two ale-pots and two bathing-pots. The sick (*i.e.*, those unable to get about at all) had fire and candle and all necessaries, *donec melioretur vel moviatur*; and one of the chaplains was assigned to hear their confessions and read the Gospel to them on Sundays and holy days, and to read the burial service for the dead. The old woman who nursed the sick and the gravedigger both received increased rations when extra work had to be done. Each leper had a yearly allowance for his clothing of three yards of woollen cloth, white or russet, six yards of linen, six yards of canvas; and on the day on which the tailor came to cut the clothes he had his meat and drink. Four fires were allowed for the whole community. From All Saints to Michaelmas they had two baskets of peat on the mess days, and four baskets daily from All Saints till Easter. On Christmas Day they had four Yule logs, each a cartload, with four trusses of straw; four trusses of straw on All Saints' Eve and Easter Eve, and four bundles of rushes on the eves of Pentecost, St. John the Baptist, and St. Magdalene; and on the anniversary of St. Martin de Sancte Crucis every leper received 5s. 5d. in money. The lepers had the liberty of seeing their friends, and strangers who came from a distance were suffered to rest in the hospital all night, but visitors from the neighbourhood departed in the evening; and when the bell sounded for supper the gates were closed. Disobedient members were punished at the discretion of the prior or prioress by corporal correction—*per ferulam modo scholarium*—and offenders who refused to submit to the usual discipline were reduced to bread and water, and after the third offence were liable to be ejected.

The following were some of the rules to be observed by lepers in the St. Julian Hospital at St. Albans: Those who were infected were to humble themselves below all other men. They were to wear a habit suitable to their infirmities—namely, a tunic and upper tunic of russet cloth, a hood and black cloak, stockings and flat shoes with upper leathers about their ankles. Those admitted to be single persons, or, if married, to part by consent and vow chastity, and if afterwards found incontinent to be expelled. To go to church regularly and continue in brotherly love. None to go beyond the bounds prescribed. None to go into the bakehouse or brewhouse. None to touch anything, because persons under such a distemper are not to handle what is for the common use of men.

The diet at St. Albans was less varied than at the Sherburn Hospital. It consisted of seven loaves a week for each leper—five of white and two of brown or black bread. Every seventh month each man had fourteen gallons of ale, and on Christmas Day each had forty gallons of ale; on St. Martin's Day each had a pig from the common herd. For some years previous to 1349 only one, two, or three of the six beds in this hospital could find leprous occupants.

